# Book Reviews

**Published:** 1819-10

**Authors:** 


### CRITICAL ANALYSIS

OF

### RECENT PUBLICATIONS, IN THE DIFFERENT BRANCHES OF MEDICINE AND SURGERY.

"I would have men know, that, though I reprehend the easie passing over of the causes of things, "by ascribing them to secret and hidden vertues and properties; (for this hath arrested and laid "asleepe all true enquiry and indications;) yet I doe not understand but that, in the practical "part of knowledge, much will be left to experience and probation, whereunto indication cannot "so fully reach: and this not only in *specie*, but in *individuo*. Yet it was well said, *Vere scire* "*esse per causas scire.*"—BACON.

***Elements of Medical Logick, illustrated by practical Proofs and Examples; including a Statement of the Evidence respecting the Contagious Nature of the Yellow-Fever.*** By SIR GILBERT BLANE, Bart. Fellow of the Royal Societies of London, Edinburgh, and Gottingen; Member of the Imperial Academy of Sciences of St. Petersburgh; and Physician to the Prince Regent. 8vo. pp. 219. London: T. and G. Underwood. 1819.

THAT a system of logical principles of a science so interesting to human happiness as that of medicine should not have hitherto been formed, by which the study of it might be reduced to methodic order, rather than, as is the case, pursued in a more or less irregular and indeterminate way according to the disposition of each individual, is a circumstance that may appear extraordinary to persons who have not contemplated this subject in a sufficiently accurate and comprehensive manner. In order to place it in its proper point of view, we should first determine what it is that constitutes the logic of a science, and how the logical principles of other sciences have been formed; for, it should be understood, physics, as well as metaphysics, may each have their system of logic.

Poetry and music present themselves as well adapted for the illustration of this enquiry. Above twenty ages have elapsed since their logical principles were instituted, and the same principles have maintained their original authority down to the present period, amidst all the revolutions of human sentiments, under the different social and political customs and habits that have intervened.

The man of pre-eminent genius, who has collected from any objects the most accurate and comprehensive series of ideas, the mutual relations of which he has also determined, has acquired a system of knowledge of such objects that must be considered relatively perfect. To trace those ideas and the order in which he has acquired and developed them, must, then, display all that is known respecting the subjects of them, and at the same time show the method by which such knowledge may be most accurately and readily obtained: it is this, we think, which constitutes the logic of a science.

From this view of logic, it must be evident that knowledge must be obtained, before the principles best adapted to direct men in general in its acquisition can be instituted. Such has been the fact with regard to poetry and music.

Homer had acquired the most perfect series of ideas of the good and beautiful, the καλοκαγαθον, of poetical sentiments, before a system of *Poetics* was formed: Aristotle only described the course taken by Homer. Euripides had impressed the mind of man with the most vivid feelings of awe and astonishment, before Longinus could have written his treatise on the *Sublime*. It was the same with respect to *Music*. The ear of Pythagoras had enabled his mind to discern the relations existing between certain sounds, before he applied the relations of numbers to them to explain the nature of harmony.

If this notion be correct, and further illustrations of it might be readily adduced, but little need be said to show why logical principles have not hitherto been instituted respecting medical science.

Our ideas of the physical, or rather the mechanical, construction of the human body, may perhaps be sufficiently accurate and comprehensive for such a purpose; but the physiology of it is yet obviously very far from being in so perfect a state; and its pathology is still less advanced, although this was cultivated in a correct manner at a very remote era. But subsequent enquirers deviated from the course taken by Hippocrates; vain researches after proximate causes were made, instead of endeavours to trace the history of phenomena; and, though at length Bacon fully displayed the folly of this conduct, it is only lately that his precepts have been followed by the generality of physicians. Of therapeutics our knowledge is far from being accurate, comprehensive, and well-determined; and it is, on many important subjects, vague in the extreme. We have discerned a connexion between certain remote results and certain agents, but we have in but a very few instances traced the progressive phenomena on which they depend. We do not mean to say, that it is to be regretted that it has not been ascertained in what manner medicinal substances primitively affect the part of the animal body to which they are applied; but that we have not discerned the order of the secondary and subsequently progressive trains of results, without which our knowledge of this subject is too imperfect to serve as a basis for any logical principles. Thus, as an example, we know not in many instances whether medicinal substances applied to the stomach affect remote parts of the system by being carried to them through the medium of the fluids, or solely by organic, or functional, connexion. Of one probably highly important subject, what may be termed *atmospheric* influence on the body, but very little is known.

The mere enumeration of these subjects, as insulated objects of study, shows, indeed, in a very forcible manner, how distant we are from that period, when a solid basis shall be laid for the construction of a system of medical logic. The separating the study of the anatomical formation of the body from that of its functions, and the functions in the state of health from those in that of disease; this, and all our histories of a multitude of individual phenomena as such, may have been useful, and indeed necessary, in a subject comprising such a multitude of various ideas: it is analogous to what has been done, apparently with benefit, in other and more confined sciences; but, even though the distinct histories of each of those subjects were rendered perfect, a system of logic could not be formed, until some mighty genius shall arise, who has the awful power of mind to comprehend the whole at one view; to trace, at the same time, the series and order of the phenomena, and to determine their relations under every variety of known circumstances. But, when may such a genius be expected to appear? The history of man shows that nations have risen, flourished, and fallen, without an Aristotle having been produced.

The hope, then, that a system of medical logic will be ever instituted, must be but very faint; or, at least, the probability of its realization must be assigned to far distant ages. This only exists for the present generation,—to proceed steadily in the true road for the acquisition of knowledge, which we have at length happily discerned. We shall thus have the gratification of employing our talents in a manner the most immediately beneficial to human nature; and we shall at the same time be collecting materials adapted for the construction of an edifice, that would constitute the most glorious and useful work ever executed by man.

Such are our opinions respecting medical logic in the most comprehensive view; but, whilst the study of the science of medicine is parted into the divisions we have enumerated, it may perhaps be allowable to consider the development of the principles of each of those divisions in an isolated state, as well as it can be effected, as the logic of those divisions: and this appears to be the opinion of Sir Gilbert Blane; for, if the work we are about to take into particular consideration may with propriety be termed "*Elements of Medical Logick,*" it can only be so in the partial manner we have just described.

In an analytical report of a work of this kind, the most clear and useful method would be, first, to give a general scope of the author's design, and then to adduce the particular illustration of his principles; but, for reasons which will be hereafter apparent, we must on this occasion commence with particulars, following the author step by step in his own course.

Sir Gilbert Blane commences the introduction to this work with a definition of medicine: he says,

"As medicine has for its object the preservation and restoration of health, it comes under the definition of an ART,—a term, the import of which consists in the adaptation of means to ends. These means must be derived from the previous knowledge of the changes producible by them, whether as corporeal agents constituting physical causes, or as affections of the mind constituting moral causes."

This we consider to be only a partial view of medicine, using that term in the comprehensive sense in which it is generally received, and in which the author has himself employed it, making it to comprise physiology and pathology, as well as therapeutics. That the two former are objects of science, must, we think, be admitted. To show this, let us consider the case of a man who has a portion of the integuments of the skull separated from that bone by a sharp-cutting instrument, without any forcible concussion of the subjacent parts: some days afterwards symptoms of disorder of the functions of the brain appear; the man soon dies; dissection after death shows signs of a diseased, and perhaps gangrenous, state of the membrane lining the skull internally, corresponding in situation with that portion of it from which the external integuments had been removed. The description of these things constitutes only natural history, and the knowledge of their occurrence cannot properly be termed science (in the received meaning of that term); but, the explanation of the relation of one of these things to the other,—that is, the explanation of the causes of the results, by showing that a due circulation of fluids in a part is necessary for its vitality; that the internal membrane of the skull is chiefly dependant on the external membrane for the supply of those fluids, which is effected by the immediate transmission of vessels through that bone from one surface to the other; and, consequently, that the separation of the external integuments from the skull, by depriving the internal membrane of the fluids necessary for its vitality, must be followed by the death of the latter. This constitutes science, just as certainly as the explaining why, in a right-angled triangle, the square of its hypotheneuse is equal to the square of the two other sides. And, though the object of this knowledge is the application of medicinal agents to the cure of disease, yet the faculty of using those agents with precision cannot be acquired without this kind of knowledge. For example, a person has a disease of the hip-joint, but no uneasiness is felt in the region of that part; the pain is confined to the knee: the practitioner, following the indications of art solely, would here apply his remedy to the

*apparent* seat of the disease in vain. Science here becomes necessary, as well as art. We think they cannot be separated in a general and comprehensive definition of medicine.

Sir Gilbert Blane next adduces a statement of what he considers to constitute reason: he says,

"The most precise criterion that can be fixed upon for distinguishing rational beings from brutes, is the faculty of adapting means to ends; and there is perhaps no operation to which the term *reason* is so appropriately applicable."

This proposition respecting reason is, we believe, generally acknowledged by philosophers; especially since the principles of it were so well displayed by MALEBRANCHE, who shows, in a very plausible manner, that *the difference between simple perception, judgment, and reason, consists in this,—that, by simple perception, the understanding perceives a thing without relation to any other thing; that, by judgment, it perceives the relations existing between one or more things; and that, in reasoning, it perceives the relations perceived by the judgment:* it is the knowledge of these relations that constitutes the faculty of adapting means to ends. But, the denial of this faculty to brutes, is a point in which many great modern, as well as ancient, philosophers, do not agree with the author. We shall pass over this question, of course, as not relating to medical logic.

The faculty of applying means to ends in the art of medicine, is a sort of knowledge that "has, to some persons of a sceptical turn of mind," the author observes, "appeared so unattainable, as not to be worth prosecuting, and they raise the previous question, *an datur ars medicinæ?*" The author argues in favour of its validity, from the actions of the brute creation, and of savages, when suffering disease: he remarks, that savages but rarely die of old age, though their maladies are fewer, and less essentially severe, than those of cultivated nations; he points out also, as an evidence of the reality of the medical art, the undoubted beneficial control it exerts over many diseases. What, too, would be the use of various plants, except as medicines? he enquires; and, finally, he says, to deny it, is to arraign the benevolence of nature, in subjecting man to the calamities of disease, without providing means for their relief. He concludes the introduction with observing, that it is his intention, "with unfeigned diffidence and humility, to endeavour to point out, in what medical truth consists; what are the difficulties that have obstructed its progress; and what the means of obviating them."

The preliminary observations to the body of the work commence with a remark, that it is the knowledge of the reciprocal relations of cause and effect, and thence the faculty of adapting means to ends, and the just application of such as we can command, which constitute skill and judgment in the cure of disease, as well as in the other arts of civilized life. "These agencies," Sir Gilbert Blane continues to state, "are ascertained by observation and experiment: by the former we may be said to listen to nature, by the latter to interrogate her." He conceives that there exists a certain relation between the structure of the body and its qualities with the surrounding natural objects and phenomena. "Every reflecting mind," the author says, "must be struck with the admirable correspondence of the structure of the living body as a whole, and of the senses and functions in detail, in relation to external nature; such as the adaption of the whole frame to the laws of gravitation, and of the eye and ear to the properties of light and air."

The author carries this sentiment further: he says there is a similar relation existing between the constitution of the mind and the laws of nature; the most essential attributes of these being the *constancy* of their operation.

"Now," he continues, "the human mind has as evident a relation to this constancy of the laws of nature, as the senses have to their respective elements; for, from the earliest period of life, there is, previous to all experience, a most unbounded confidence in the present and future constancy of events, manifested in all the actions and attainments of practical life. The belief that the sun will continue to rise every morning; that all bodies will continue to gravitate to the earth; that the human beings around us exist, feel, and think, as we do, may be quoted as examples of this untaught knowledge."

If this opinion be correct, the fable of the Sybarites, who blinded themselves in the darkness of the first night, believing their eyes would in future be devoid of use, must be considered an idle invention, instead of being, as the doctrine of some great philosophers would lead us to interpret it, a symbolic illustration of a metaphysical truth.

Sir Gilbert Blane, proceeding in a train of reasoning on the same principles, says,

"But this is not all, nor the most important coincidence of the frame of the mind with the established course of nature. In all the effects produced by the action of external bodies on each other, and on our own bodies, there is a rapid and instinctive connexion established between cause and effect, in virtue of that part of the structure of the mind by which it is made susceptible of habit and association, particularly in early life. So that, not only every organ and function of the body, but every faculty of the mind, is co-relative with, or represents and reflects, as it were, the elements and laws of universal nature."*

* "See this sentiment more fully illustrated, in a lecture on Muscular Motion, read before the Royal Society, by Gilbert Blane, M.D. page 40. London, 1789. It is also most ingeniously and appositely alluded to, in Madame de Stael's account of the German poetry, in her work entitled *De l'Allemagne*, page 334, vol. i. Paris, 1815."

This doctrine was first advanced by the author in a discourse read to the Speculative Society of Edinburgh, in the year 1771; and, as must be obvious, it is in opposition to that which states custom to be the only source of our ideas of cause and effect. It was against Mr. Hume, the most forcible advocate of that doctrine, that Sir Gilbert Blane especially made this attack. We shall not presume to adduce our opinion on a question that still divides many great metaphysicians; but we must remark, that we think Sir Gilbert Blane, in the following paragraph, rather favours, than controverts, the notions he opposes. He says,

"These confident expectations of the future, could never have been discovered by reasonings *à priori;* inasmuch as we know nothing of the tie which connects cause and effect; *nor can we form any anticipations of future events, but from the past experience of what may be called simple sequence.*"

These speculations must not be considered totally foreign to the proper subject of this work, because, by reasoning directly or analogically from what we have been taught by custom; or, according to the doctrine of Sir Gilbert Blane, by finding that, in imitating the sequences of nature, we can adapt means to ends, so as to bring about certain results; we acquire our first idea of *power*,—the want of accuracy in which, in the forcible expressions of the author, "has given rise to those mischievous errors and inveterate prejudices, those numberless fallacies, those nugatory and superstitious practices, with which the history of the world abounds, and which have proved sources of vice and misery, embittering and deforming human life and conduct."

We shall not pursue this subject further than the author has here done; we think, with him, that some attention to it forms a proper introduction to medical logic: but it is from treatises expressly metaphysical that the knowledge of it must be acquired.

To determine correctly the relations of causes and effects, and from first and real principles to form rational inductions, is, as the author observes, the true object of philosophy. The living body being endowed with such a multitude of properties, and, consequently, its functions being so numerous and so variable under different circumstances, cannot, without extreme difficulty, be submitted to this rational research.

"But," Sir Gilbert continues to observe, "it is incumbent on those who allege that there are obstacles to physiological investigations, seemingly so insurmountable, to specify what they are.

"The author, therefore, submits to the profession the following enumeration of the properties peculiar to animated nature; meaning under it to describe all the ultimate facts, or primary elements, which form the ground-work of physiological and pathological science.

"These energies may be arranged as follows:

1. The Generative.
2. The Conservative.
3. The Temperative.
4. The Assimilative.
5. The Formative.
6. The Restorative.
7. The Motive.
8. The Sensitive.
9. The Sympathetic.

"This arrangement differs from any with which the author is acquainted, inasmuch as it is not founded on an enumeration of functions and organs, but on elements pervading and belonging to the whole animal system. It is meant to comprehend all the properties in which the essence of life consists, and which characterize and distinguish it from inanimate matter on the one hand, and from a moral and intellectual nature on the other."

Had the author not expressly stated, that the above arrangement of the "primary elements of physiology" was not founded on an enumeration of functions, we could not have considered that he had intended to adduce any thing at all novel. Every system of physiology treats of the generation of the body, of the causes of its temperature, of the assimilation of the food, &c.; but these things are regarded as the results of functions.

We have exerted much painful thought in endeavouring to understand the author's precise meaning, and we are not certain that we have discerned it; for his illustrations of his principles rather embarrass than assist us. In the above paragraph, he speaks of certain energies existing in the body, of which we may suppose the functions usually designated by analogous substantive terms are the results: to assist the conception of the reader, we will term them so many *archæi;*—but, on defining them, Sir Gilbert Blane on some occasions gives a substantive definition of the results of functions: thus, he says,

"The Assimilative.—This consists in the chemical changes brought about in the decompositions and combinations effected by the power and processes peculiar to life."

"The Temperative.—By this is meant that steady degree of heat with which all animals are endowed."

And, in another place, after noticing the hypotheses that have been advanced respecting it, he says,

"On weighing the whole evidence, it seems clear, that the heat of the living body is chiefly generated by the vital energies."

Thus, then, the temperat*ive* principle is *heat;* and heat is the result of the play of the vital *energies*. This word, in this place, can only be another term for *functions*.

"The Motive.—By this is meant muscular action, in its most extensive sense."

"The Sensitive.—Sensation, being a simple idea, does not admit of definition, &c."

Others he defines, certainly, as active principles or primary agents: let us take a view of his dissertations upon these. We transcribe the whole of what he advances respecting the first:

"The Generative.—It will not be disputed, that this primary energy of nature belongs purely and peculiarly to animal and vegetable life. Being emphatically named the mystery of nature, and being now admitted, by all correct physiologists, to be inexplicable, it only requires in this place to be barely enunciated. It may not, however, be without use here to hold out as a beacon to those who may still be disposed to waste their time and labour in attempting to overleap the stated boundaries of nature, the fruitless and absurd results they are likely to attain; for, what can be so extravagant and irrational, as that hypothesis which professes to explain generation, by supposing an infinite involution of embryos: *Obscura obscurioribus*. The doctrine of that most respectable physiologist, Dr. Blumenbach, who refers generation and growth to what he calls the *formative nisus*, is perfectly consistent with reason; inasmuch as it is to be considered rather as an exposition of facts than as a theory."*

We shall follow the author's judicious advice, and not waste time in enquiries whether generation should be considered as the result of the play of certain functions of organized bodies, or as the work of an intelligent energy, an *archæus*, within organized bodies, moulding animal matter at its will; particularly as the subject of discussion is *medical logic*, on which nothing doubtful should be advanced,—nothing that is not an exposition of observed facts. Though, we must say, it is somewhat curious that we should be cautioned against enquiries on this subject, since they are likely to lead to absurd results, and immediately afterwards the doctrine of a physiologist designated as perfectly consistent with reason: whether as an exposition of facts or as a theory, does not signify; because, if one man has been able to discern facts respecting it, another may hope to do the same to a greater extent: and what is any knowledge but ideas of facts, or any theory but an exposition of them?

We proceed to the consideration of the author's illustrations of the other energies.

"The Conservative.—By this is meant that power by which the living body is prevented from running into putrefaction. According to the experiments of Dr. Alexander,† the range of temperature most favourable to the putrefaction of dead animal matter, being between

* "See D. I. F. Blumenbach *de Nisu Formativo*, Göttingen, 1787; and *Abhandlung über die Nutritionskraft*, St. Petersburg, 1780."

† "See *Experimental Enquiry on the Causes of Putrid Diseases*, London, 1771."

86° and 100° Fahrenheit, includes the usual standard of animal heat: there must therefore be some powerful energy in life itself, which counteracts this tendency to spontaneous decomposition."

Reasoning on this must be hypothetical;—it is certainly hypothetical to suppose the existence of an abstract intelligent agent in organized bodies, for the sole purpose of preserving them from putrefaction: but it is a very probable hypothesis which attributes the preservation of living animal bodies from putrefaction, to the affinities between its particles constantly in play whilst its functions are performed. When these cease, those bodies then become subject to the more powerful affinities of the surrounding medium, which are chemical affinities, whilst the particles of the dead body are kept together only by the common gravitation, and the weak cohesion of soft substances.

The author, referring to this opinion, which was that, he says, of Dr. Alexander, and some other physiologists of that day, (and is that of many great physiologists of the present, too, should have been added,) says, "It is quite inadequate to account for this striking phenomenon; and that there is an antiseptic power in life, independant of motion and the change of matter, is proved by the same principle of self-preservation being found in the quiescent state: for instance, in impregnated eggs and torpid animals."

These things afford no support whatever to the author's hypothesis; for it is certain that torpid animals are *not in a quiescent state;* and it is very probable, if abstract reasoning alone can make any thing probable, that the egg is not so. The statement we have made respecting torpid animals, has been satisfactorily proved by the experiments of Professor MANGILI, of Padua: he has ascertained that respiration, and of course the circulation of the blood, are slowly carried on. This might have been well supposed; for, if there were no actions going on in them, how is the fat with which they abound before they become torpid absorbed, leaving them comparatively thin and emaciated when they come out of that state?

And, how can the egg be supposed to be in a quiescent state? It contains an organized mass: how can this have been formed without action? How is the chick subsequently developed, without action? It is more rational to suppose that a degree of action, insensible to our eyes, is constantly going on in the living egg, analogous to that by which its organization was developed,* than that it ever totally ceases for a time, and is then resumed. Besides, why is a living egg warmer in a certain

* MALPIGHI says, the body of the chick is formed in an egg before the incubative heat is applied.

temperature of the atmosphere than a dead one? and why is it not frozen in the same degree of heat? How can the *materia vitæ*, the existence of which the author favours, cause this difference in the living egg, in any other way than by producing *action*? We are at a loss to conceive how an organized mass, in an absolutely quiescent state, can be said to be influenced by any agent. If it is influenced by it, it must be acted on by it, and re-action must ensue. If it is not influenced by it, we must look for other reasons for the phenomena we have alluded to; and no reason can be so satisfactory, considering the subject in an abstract manner, as that there is a series of actions going on; and analogical reasoning from observed physiological facts shows the probability of this opinion; always bearing in mind that an egg is an organized animal body: and, with respect to the want of evidence to the senses of the existence of action, living plants stand in a similar relation to them.

The author adduces some other general arguments, which we consider as still less valid than those we have noticed, in favour of the existence of a *materia vitæ*, the original invention of which doctrine he attributes to John Hunter; and he expresses his surprise that "we meet with works on physiology, some of them even professing to be complete systems, in which this fundamental law of life is not alluded to."

Here we do not comprehend what the author means to signify: terming the existence of a principle, supposing it were not suppositious, a *law* of life, is using language in a manner so different from its common acceptation, that we cannot at all understand it.

We must make some remarks respecting the neglect of this doctrine of the existence of a *materia vitæ*. It is not admitted, either because it is considered that the cause of *life* cannot be regarded in an abstract manner, as distinct from organization; or because the admission of such a principle is, at least, contrary to the mode of philosophizing inculcated by Bacon.

We here pass over the *assimilative* and *temperative* energies, because, as we have already shown, the author defines them, substantively, *heat* and *assimilation*, and states these to be results of the *functions of the* system.

"The Formative.—This may be called also the organizing or plastic. It has not usually been stated as a principle distinct from the last, (the *assimilative.*) The slightest reflection, however, must evince that it is quite a separate act of nature, and as different from the assimilative as the construction of an edifice is from the preparation and collection of its materials."

It is by this, the author says, that the processes of growth and repair are effected; and, as a subject for wonder, "with such harmony on both sides the body, as to produce that correspondence and symmetry which we behold!"

"This is a subject," the author continues, "the nature of which eludes the keenest research, and overwhelms the mind of man with astonishment and despair; from which it can find no refuge, but in resting on it as an ultimate fact, and referring the whole to supreme intelligence."

In the latter sentiment we most cordially concur, but not in the former of those contained in the foregoing paragraph. We have, happily for our mental comfort, ceased to feel despair at not being able to discover the proximate cause of any natural phenomenon; but we know what it is to experience this suffering, and we yet remember it with pain. In early life, before we had studied the works of BACON, or become acquainted with the disputes of the Academic sect, as may well be supposed, we endeavoured to discover the cause of apparently one of the most simple of natural phenomena,—the passing of a ball from the hand of a person in a direction different from that which it would take when solely influenced by the laws of gravitation. Our books told us that *momentum* was given to it. But what is *momentum?* Is it something material, or is it immaterial? and how can it act on the ball after this has left the hand? We were indeed "overwhelmed with astonishment and despair." The hints these remarks convey may prove useful to some of our younger readers.

Returning to the author's dissertation on the formative energy, we find him remark, in continuation from the paragraph last transcribed, that,

"Should any one attempt to scan it further, by ascending higher in the scale of natural causes, (higher than supreme intelligence?) he will either find himself baffled, or will be in hazard of falling into some extravagance; such as that of Van Helmont, who held that there was in living beings an intelligent principle, which he called Archæus, presiding over, and directing, the secret movements of the animal machine; or of Stahl, who referred it to the rational soul."

We revere VAN HELMONT; and regret to see his opinions marked with the stigma of blame, without their merits being at the same time designated. His accurate and penetrative views led him to discern that there must be an active and powerful influence, seated about the region of the stomach, distinct from the other chief influences regulating the functions of the system; that is, distinct from the brain and spinal marrow. The functions of the ganglionic system of nerves had not then been determined by experiments; this was not effected until nearly a century after, by JAMES JOHNSTONE: but Van Helmont had in a manner anticipated the discovery; and the making that influence consist in an intelligent immaterial principle, was, with a few chemical absurdities, as Sir Gilbert Blane indeed observes, the error of his age. Put the ganglionic system of nerves in the place of his *archæus*, and his works must be perused with benefit and delight. We proceed with the transcription of another paragraph:

"The proper function of the formative faculty, is growth and repair. The long and universally received mode of conceiving the progress of growth, was that of a constant accretion of organic matter, giving additional length and breadth to the parts nourished. But it is evident that this would render the preservation of shape utterly incompatible with the enlargement of dimension; and it was first clearly demonstrated by Mr. John Hunter, that the only process by which the growth of solid parts, particularly bones, could be carried on, was by a constant removal and replacement of particles."

Here we must correct a little error in the history of physiological opinions. It may be difficult to determine when the nature of this process was first demonstrated, because analogous notions were advanced by the ancients; but it is certain that it was well developed long before the time of John Hunter. The story of the Dutch advocate, who saved his client from the sentence of the law for having committed murder nine years previously, by bringing distinguished physiologists to state their opinions that the human body was totally renewed in less than seven years, and therefore, as he showed, his client could no longer be the same man, is very ancient. However, this doctrine is fully and accurately displayed by Bordeu; and we particularly notice this, because we can at the same time refer to an author who dwells with much earnestness on some pathological notions, which Sir Gilbert Blane also advances as worthy of consideration: that there seem to be particular outlets for particular species of *effete matter*. Bordeu has perhaps carried this doctrine to excess, in his endeavours to rescue the physiology of the human body from the dominion of the chemists and mechanists, in that curious specimen of fervid discussion, his *Analyse Medicinale du Sang*.

On the *restorative energy*, the author observes, "it is well remarked by Dr. Gregory,* that it carries in itself the means of repairing the injuries and disorders incident to it." The chief illustrations of this which the author adduces, are the phenomena attendant on sleep; several very curious and interesting facts respecting which he adduces; and its agency, as the *vis medicatrix naturæ*.

Whether the phenomena last alluded to depend on an abstract intelligent principle, or are results of the common laws of

* "See *Conspectus Medicinæ Theoreticæ*, vol. i. p. 5."

the animal economy, is a question likely to be a subject of scholastic disputation for a long period to come. We shall certainly not attempt to discuss it, whilst the works of Boyle and Glisson are in existence.

The *motive*, the *sensitive*, and the *sympathetic*, energies, we shall here pass over; because, as we have already observed respecting the two former, the author defines them as results of the functions of the system.

Here we arrive at the termination of the first section of this work ; and we repeat, we are by no means certain that we have seized the precise meaning of the author respecting the "ultimate facts, or primary elements, which form the groundwork of physiological and pathological science," not founded on an enumeration of functions. We have some conception that he may intend, by these energies, only to designate phenomena resulting from the exercise of the functions, but which are not themselves the functions: in other words, that these are an analysis of the functions. But then, his express language does not authorize such a supposition ; on the contrary, it, with regard to some of them, designates those energies as species of abstract intelligent agents existing in the animal body, each having its peculiar office, and exerting its influence on animal matter in virtue of its will, if it may be so expressed. And, if by these energies it is intended, as we have supposed, to designate an analysis of the functions, then there is nothing whatever of novelty in what the author has adduced, as far as relates to *principles ;* though his illustrations of them, regarded in the point of view last alluded to, are often original and ingenious.

No work ever passed our critical examination that we have been so desirous to press on the perusal of our readers, as the one before us. The reason of this must be too obvious to require explanation.

[*To be continued.*]

---

***An Inquiry into the Nature and Properties of the Blood, as existent in Health and Disease.*** By C. Turner Thackrah, M. Roy. Col. Surg. and Lc. Soc. Aph. 8vo. pp. 132. London : Cox and Son. 1819.

Est quodam prodire tenus, si non datur ultra.—HORACE.

This work has been produced, the author states in a preface, in consequence of Mr. A. Cooper having offered to the gentlemen educated at the school of Guy and St. Thomas, a prize for the best dissertation on the Blood. "I instituted some experiments on the subject," he continues to observe, "and stated the results which my observations afforded. This essay being so fortunate as to obtain the prize, I have been induced to present it to the public: and, since the late period at which I heard of Mr. Cooper's proposal prevented the Inquiry's comprehending, in the first instance, some points of importance, I have, during the last year, been endeavouring to supply the deficiency."

We have not often perused a work to which we could give more unqualified approbation. Extensive and sound erudition, great accuracy in all the necessary observations and experiments, comprehensive views, and a particular clearness of exposition, mark the character of the work; and, what is of especial importance, and which renders it interesting beyond the merely physiological relations of the subject, the author has seized every opportunity of making researches tending to the improvement of the practice of medicine; and he has adduced from them several novel and highly-useful observations, which he has illustrated by his judicious remarks.

The circumstances of this being a successful prize-dissertation, and the subject being one respecting which a new and comprehensive treatise was so much required, deduced from researches after facts, uninfluenced by any preconceived hypothesis, will, we feel confident, induce medical practitioners in general to become possessed of the work: we shall therefore pass over in a rapid manner the chief part of it, that we may notice more at length that which is especially applicable to the practice of medicine. An abstract of this may prove a fertile source of reference, on various important occasions.

After some general observations on the origin, character, and uses of the blood, the author notices its most remarkable physical characteristics: and, first, its coagulation on removal from the body, and its change from a homogeneous fluid to the division into the serum and crassamentum. Its proportion to the weight of the body, is next considered. "Keil," the author observes, "estimated it at one hundred pounds; others do not believe it to exceed eight pounds. Haller computes it at ten; Young at forty; and, in Cooper's lectures, its proportion to the solids of the body, is considered as one to sixteen or twenty." Eight or ten pounds, he justly remarks, must be concluded too small a quantity for the supply of the numerous vessels of the body, occupying so extensive a space; and, indeed, it seems to be shown by the quantity lost within a few days, spontaneously or abstracted by art, being frequently greater than that, without the destruction of life.

The question of the vitality of the blood, is next briefly discussed; which the author is disposed to decide in the negative. The arguments he adduces to this effect, are novel and ingenious. The advocates of the affirmative are, we believe, becoming daily less numerous.

On the subject of the chemical qualities of the blood, no original observations are adduced.

The next chapter is on the peculiarities of the blood in different classes of animals. The author first confirms the statement of former physiologists, that there is but little variation of its character in those of the higher orders. The *relative quantity* was found, generally speaking, to be less in birds, fishes, and the weaker animals, than in the larger and more muscular. No uniform disparity was found to exist in the *relative quantities of the serum and crassamentum;* but, the author states, "it appears probable, that a more complete examination would prove the crassamentum to bear a proportion to the strength and ferocity of the animal." With respect to the *periods of coagulation*, "a general inference might be drawn, that coagulation commences sooner in small and weak animals, than in the large and strong." Its *temperature* in the horse, when flowing from a vessel, is 97°; in the ox, 100°—101°; in the sheep, 102°—103°; and in the duck, 107°. This, the author remarks, is conformable to the statement of Braun. No considerable variety exists in its *chemical qualities;* excepting that, according to Berzelius, that of the ox contains a smaller quantity of saline matter, and a larger proportion of azote, than that of man. Its *specific gravity* is nearly uniform. "The *red globules* exist principally in the more perfect animals: in the mammalia and birds, partly in fishes; but not generally in reptiles, insects, and worms. In some creatures, coloured blood is found in the vessels near the heart, while the rest of the body is supplied only with a serous fluid." "Of those creatures which want the red particles, most have *white* globules; but, in the lowest orders, even these cannot be discerned by the microscope." The blood of some animals is found, while circulating, to contain air-bubbles. In the land and sea tortoises, in some fish, in the hedge-hog, and the viper, this appearance has been asserted by respectable writers.—MORGAGNI, Ep. v. 22.

The *coagulation* of the blood is then particularly discussed. On this subject we find some original observations, possessing a considerable degree of interest. The author first notices the effects of chemical agents mingled with it on removal from the body. Medicines, internally administered, had no apparent influence on it. Stupefaction from opium does not affect it. It is induced readily, in proportion to its paucity. Agitation retards it. With respect to temperature, it concretes soonest at from 100°—120°; next at from 40°—50°; and last, and with a greater disparity, in that of from 60° to 90°. And the author thinks it worthy of remark, that the serum is most readily and copiously effused in the higher temperatures; and this, he believes, in regular gradation.

A subject which particularly engaged the author's attention, is *the comparative periods of coagulation, as influenced by the strength or weakness of the vascular action.* From various and repeated experiments, under different circumstances, on the blood of oxen, sheep, horses, dogs, and swine, it appears that the *blood coagulates slowly, in regular proportion to the tonic state, or that condition of the system in which the vital powers are strongest.* It also appeared, that coagulation occurred soonest in *venous* blood.

The *causes of the blood's coagulation* constitute one of the most interesting parts of the enquiry; and it has been investigated by Mr. Thackrah with care and attention proportionate to its importance. We shall only adduce his conclusion respecting them; remarking, at the same time, that his grounds for it appear to be satisfactory, though we are unable to form any rational explanation of this phenomenon. It is "that the vital or nervous influence, is the source of the blood's fluidity; and its loss, the cause of coagulation." But this conclusion, it must be observed, applies to the vessels in which it is contained, not to the fluid itself. Indeed, the series of experiments that led the author to form this conclusion, appear to us to furnish very powerful arguments *against* the doctrine of the vitality of the blood.

The above opinion of Mr. Thackrah, we should remark, is similar to one advanced several years since by Mr. Charles Bell, in his Anatomical Lectures; and was then, we believe, considered to be merely visionary by many of his auditors. But the experiments of Mr. Thackrah seem almost to demonstrate its truth.

We now arrive at the chapter treating of the *changes produced by disease.* After some reflections on the degree of importance and certainty attached to them, the author treats of the *quantity* of the blood. This is a subject on which only probable suppositions can be formed, but they are in favour of its diminution under certain circumstances. The *colour* of the blood is frequently altered in a remarkable manner. Long-continued hæmorrhages render it pale. Sometimes, chiefly observed in robust and muscular men, the venous blood is of a dirty-red colour; and it is also occasionally found to be florid. A darker, or livid, hue of it is stated by authors to have been witnessed in *malignant* fevers. Several instances are related, or referred to, when it has been found so much lowered in *temperature,* as to be cold to the touch; and this without the patient apparently labouring under any severe disease. The relative rapidity of its *coagulation* has been already adverted to, and the cause of it explained.

"*The firmness of the coagulation of blood*," the author observes, "has been considered a distinctive mark of the tonic state of the system; its greater tenacity, a characteristic of inflammation; and its looseness, a sure proof of debility?" Although these marks cannot wholly be relied on, yet his experiments have shown that they are in general indicative of the states of which they are said to be signs. Much difference in its appearance will arise from the size of the vessels in which it is received. "The fluid of blood received in a bason, is usually in greater proportion than that contained in a small cup; and, of course, the cake in the latter is looser than that of the former." And, as a general practical axiom, he says, "If, on the division of the coagulum, at the expiration of from eight to twenty-four hours, there ensue no considerable effusion of serum, and the crassamentum remain extraordinarily firm, I believe that further depletion is fully warranted." He next adduces the results of the experiments of Dr. Langrish, and the observations of Dr. George Fordyce, respecting the tenacity of the coagulum in different diseases; from which it appears, that in acute maladies it is generally inordinately dense.

"We frequently," the author proceeds to remark, "observe much benefit derived from bleeding, even when the crassamentum is soft and yielding; nor should we, in such cases, hesitate to repeat the depletion, if other circumstances indicate its propriety. Dr. Watt, in his cases of Diabetes, remarks, that great advantage accrued from venesection, though the coagulum was loose and black; and that, on repeated evacuations of blood, the crassamentum became much firmer, and of a more natural hue."

Here, of course, blood-letting produced those changes, by removing the diseased state on which the alteration of the qualities of the blood depended; for such a condition of the blood is not common in diabetes. It is usual to find it present the sizy coat in that disease.

The *proportion of serum and crassamentum*, is next considered. This is usually in a ratio *inverse* to the strength of the system. Two very interesting observations are adduced, which show that the *serum is relatively increased during the continuance of bleeding*, and in a considerable degree in the space of a few minutes. Dr. Weatherhead suggested the following query to the author respecting this circumstance;

"Does not the abundance of serum in the last-drawn cup, arise from the immediate effect of the bleeding, in rousing the energies of nature to absorb serum from the different cavities, and thus occasion the fact you have remarked? If this be the true cause, it will likewise account for the benefit derived from, and authorize, the detraction of blood in both sanguineous and serous extravasations, wherein the strength has not thereby been materially diminished. This absorption ought also to be more obvious in health, where nature acts unfettered by diseased associations."

The circumstance must, Mr. Thackrah conceives, originate either in the cause Dr. Weatherhead suggests, or in the greater disposition to concretion which the blood assumes, when the system is reduced. "The latter opinion," he continues, "is the least probable; for, if the increased proportion of fluid in the last-received blood, arise merely from the speedy contraction of the crassamentum ejecting a greater quantity of serum than the first-drawn, this substance would be firmer in the last than in the first."

The *mechanists* would readily explain the above phenomenon by the *vacuum* produced in the vessels by the abstraction of blood; whilst the *animists* will perhaps concur in the opinion of Dr. Weatherhead. Others will seize the fact as an important indication for practice, without attempting to form any conclusion respecting its cause.

The author next adduces a statement of the relative quantities of the serum and crassamentum, in various forms and stages of disease; which is consonant with the general account already given.

We now arrive at another very interesting part of the subject, —*the coat of yellowish size, which sometimes covers the surface of the blood.* We cannot extend the limits of this analysis much further; and therefore, to avoid repeated particular remarks, we state, that the whole of the observations and experiments contained in this chapter, tend to show the truth and importance of the opinions of Bordeu, given in the last number of this Journal. We hope to find them become the subject of discussion, and shall therefore at present briefly pass over this part of the enquiry.

Mr. Thackrah satisfactorily shows, that the sizy coat *does not arise from any particular tenuity of the blood,* as stated by Hewson; and this physiologist proved, that *increased gravity of the cruor was not* the cause. And though the author is disposed to infer, that the formation of the buff-coat in inflammatory diseases depends on the blood's indisposition to coagulate, such tardiness of concretion allowing the red particles to subside, yet he acknowledges that an observation of Dr. Whiting militates against this opinion. "He found a fibrous tunic near the *bottom* of the crassamentum in one case, and this phenomenon to be wholly absent in another, though the blood remained fluid from fifteen to thirty minutes." We cannot here pass over one circumstance that we have ourselves almost constantly observed, and in instances of which the works of every writer on inflammatory affections abound; which is, that the buffy coat is not witnessed in the first or second day of the disease, yet it afterwards appears, even though the severity of the malady has become much alleviated. The observations and experiments of Mr. Thackrah, in regard to this part of the subject, are not quite perfect; since he has not noticed the stage of the disease in which the blood forming the subject of them was drawn.

The author has also been led to doubt the truth of the opinion, attributing the buffy coat to *increased agitation of the blood.*

Mr. Thackrah adduces the following queries respecting it; the analogy of one of which to some opinions we have already noticed will be easily discerned.

"Does the additional quantity of fibrine, acquired by inflammatory blood, remain for some time unassimilated, and thus retard those changes which it is natural for this substance to assume?

"Or, rather, shall we believe that the vital energy is preternaturally excited in active disease, and thus, by its effects on the blood-vessels, protracts the period of coagulation?"

It must not be forgotten, that there is an increase in the relative quantity of the fibrine to the serum, *in the latter stages of most acute diseases;* and, also, that the sizy coat appears in diabetes, when the vital energy is much depressed, and when the blood is not in a state of inordinate tenuity.

We pass over several judicious remarks relative to the application of those facts in the practice of medicine, to the consideration of the *changes produced in the quality of the serum* by disease. On this subject Mr. Thackrah adduces some interesting observations from other authors, but nothing novel of much importance. The same remark will apply to the subject of *depositions from the blood* in disease.

That there is a disposition to *putrescency* of the blood in many diseases, is satisfactorily proved, in spite of the scepticism and objections of those who *absolutely* abandon all considerations of a humoral pathology. This *putrefactive process* Mr. Thackrah believes to be influenced by the state of the system. He says, "It takes place most readily in a debilitated condition. Blood subtracted during inflammation, assumes this change later than blood during weak vascular action." This would lead us to attribute it to the same laws that regulate the coagulation of the blood.

The rapid and partial analytical sketch we have here given of the work of Mr. Thackrah, is produced, as we before observed, rather from a desire that our Journal should comprise some of the valuable matter it contains, than from an attempt to convey, in an abstract, any thing like an appropriate idea of its contents.

*The Influence of Civic Life, Sedentary Habits, and Intellectual Refinement, on Human Health and Human Happiness; including an Estimate of the Balance of Enjoyment and Suffering in the different Gradations of Society.* By James Johnson, Esq. Surgeon to his Royal Highness the Duke of Clarence; Author of the "Influence of Tropical Climates on European Constitutions," of a "Practical Treatise on Derangements of the Liver, Digestive Organs, and Nervous System," and Editor of the "Medico-Chirurgical Journal, or Quarterly Register of Medical and Surgical Science." 8vo. pp. 93. T. and G. Underwood, 1818. With the epigraphs,

Et mores hominum multorum vidit et urbes.—VIRG.

He studied from the life,
And in the original perused mankind.—ARMSTRONG.

In this discussion on the balance of enjoyment and suffering in the different gradations of society, the author, already so well known and favourably regarded by the profession, has adduced, in a concise form, many interesting and important observations and reflections respecting several of the most severe diseases of the animal economy; especially those of the gastric system, the heart, and the brain.

One of the most marked characteristics of the whole of Dr. Johnson's works, is derived from a view which he has taken of the system, that, if Darwin be excepted, had until lately been but little attended to by English pathologists. He regards the human body as a system of organs connected together by nervous systems, so that an impression cannot be made on any part without being more or less experienced over the whole; the phenomena consequent on which constitute what are termed sympathetic affections, and these often become the most serious of the diseases offered to our observation. Another thing is, that, under ordinary circumstances, the animal economy possesses a certain and uniform degree of vitality, which cannot be increased in one part without being relatively diminished in another, which always happens in disease, the cure of which, on most occasions, must consequently consist in restoring the due degree of vital action, by stimulants, to the parts that have lost it, when this can be done; or else by inducing counter-irritation in a part where its presence will not be attended with danger, and which, by sympathy, will remove it from more important organs.

Now, although this doctrine is not original with Dr. Johnson, yet he has the merit of having displayed it with accuracy and precision, and of having discovered many of its laws. This is shown in his works on Tropical Climates and on Atmospheric Influence. We find, too, much interesting matter respecting it in the work before us. It would not be possible to give an adequate view of it in an abstract, and the work itself is drawn up in so concise a manner, as not to admit of an analysis occupying much less space than the original; we shall therefore confine our account of it to two extracts, illustrating, respectively, the two latter points of doctrine to which we have just alluded.

"The ploughman, exposed at all seasons to the inclemencies of the skies, and strenuously exercising his voluntary muscles, might gormandize with safety on alderman's fare. But not so the citizen, however well-trained in the school of Epicurus. His sedentary life, and a host of moral and physical circumstances around him, render it a matter of impossibility that *repletion* shall not succeed even an apparently temperate regimen; and, in reality, this repletion, and the irregular states of plethora which thence result, characterize nine-tenths of the diseases of civilized life, though they assume the garb of debility, and too often lead to the most erroneous and unsuccessful methods of treatment. Every one, after a full meal, especially of animal food, with all the *et ceteras* of a civic table, must have felt how incapacitated he was for either mental or corporeal exertion. It is a law, indeed, in the economy of the living machine, that, where any one of the three systems above mentioned (the organic, the animal, and the intellectual,) is over-excited, one or both of the other two systems must fall into a state of irregular or deficient action. The heavy meal of animal and other food exemplifies this law: when the digestive organs and circulating vessels are strongly engaged, the muscular and the intellectual systems are indisposed towards the full exercise of their functions, the greater portion of vital energy being then apparently concentrated in the *organic* system, the principal theatre of operations for the time. On the other hand, let the animal system, or voluntary muscles, be thrown into violent or unusual action,—the digestive process is diminished, or even suspended; and the mind is incapable of dwelling intently on any train of thought. Who could solve a mathematical problem immediately after a furious cricket-match? Again: let a man sit down to an intricate calculation, or the investigation of an abstruse literary subject; nay, even to the perusal of an interesting poem, or other effusion of genius; and the appetite will be so withdrawn, that the hour of dinner will be scarcely remembered."

"I have shown, that in civic life, as now constituted, the digestive organs are very generally in a state of *irritation*, from the quantity and quality of our food, drink, &c. The situation of the *nervous system* will hereafter be proved to be similar. To remove these evils, man will not avoid the causes that produced them; the only alternative, then, is recourse to medicine. But almost all medicines are in themselves *irritants;* and more than half the employment of the physician consists in removing one irritation by inducing another. Let us exemplify this remark. A man, after full living, sedentary avocations, and irregular hours, begins to feel loss of appetite, headach, drowsiness, depression of spirits, fickleness of temper, with sense of fullness, and uneasiness on pressure in the right side, &c. There is now engorgement and *irritation* in the liver: what do we do? We give calomel, aloes, and colocynth, which *irritate* the mucous membrane of the digestive organs, stimulate the mouths of the biliary ducts, and cause a flow of bile and various other secretions into the intestines; which secretions are soon carried out of the system entirely. The whole train of symptoms now vanish, like a fog before the sun-beams. But suppose (which indeed is every day done) we had employed a different class of irritants, called tonics, as steel, bitters, &c. which the loss of appetite and other symptoms would *appear* to indicate? Why, the result would be an aggravation, in the end, of all the complaints. Hence, then, we perceive that nothing but the most careful and minute investigation of the *nature and seat* of the morbid irritation, can enable us to apply the *artificial irritation* of medicine, with any prospect of ultimate success. This view of the subject might open the eyes of mankind to the devastation which is daily produced in the digestive organs, by the careless and indiscriminate administration of a farrago of medicines, which, like food and drink, both by their quantities and qualities, keep the whole line of the alimentary canal, and in fact the whole system, in a state of morbid irritability."

---

*A Treatise on the Physiology and Diseases of the Ear; containing a comparative View of its Structure and Functions, and of its various Diseases, arranged according to the Anatomy of the Organ, or as they affect the External, the Intermediate, and the Internal, Ear.* By JOHN HARRISON CURTIS, Esq. Aurist to his Royal Highness the Prince Regent, his Royal Highness the Duke of Kent, their Royal Highnesses the Duke and Duchess of Gloucester; Surgeon to the Royal Dispensary for Diseases of the Ear; Lecturer on the Anatomy, Physiology, and Pathology, of the Ear; Fellow of the Medical Society of London, &c. &c. 8vo. pp. 136. Second edition, with considerable additions and improvements. London: Anderson and Chase, 1819.

SOME account of the first edition of this work has already appeared in a former number of this Journal; but, from the extracts there adduced conveying an inappropriate idea of the contents of the present edition, we are disposed, from sentiments of justice towards the author, to notice it again on the present occasion. We shall, in the first place, give a general account of the subjects treated of in it; and then make some remarks on the merit of the practical observations it contains.

Mr. Curtis commences with some reflections on the benefit which has accrued to the medical art, from practitioners devoting their attention exclusively to the diseases of particular organs; and, when this has been done after due study of those of the general system, as is the case with the author, this remark must be acknowledged to be correct. Some observations on the function of hearing in its intellectual relations, and the consequences of the want of this faculty, then follow; and this leads to the consideration of the state of persons in whom it has been connate, and on the probability that medical means might be employed with success in relieving many cases which have frequently been considered to be irremediable.

After a reference to the different authors who have treated of the anatomy and diseases of the ear, Mr. Curtis gives a description of that organ, with some illustrative observations deduced from comparative anatomy; this is necessarily connected with some physiological remarks, and with a brief account of the the theory of phonics, as connected with its functions. This leads to the consideration of its diseases. On this subject we must receive with particular pleasure any thing calculated to lead to improved modes of treatment, or to remove that apathy and prejudice against resorting to medical assistance, to which the patients of this affliction have so generally given way. This may to a certain extent be well-founded, from a considerable proportion of cases being essentially without the limits over which the medical art exerts its successful influence; but it is certain that many cases are susceptible of relief by judicious treatment: the grateful acknowledgments of the public, and the assistance of the profession, are, we consider, therefore due to those surgeons who devote themselves to this neglected and unattractive subject of pathology. Perhaps the disposition now prevalent to seek for the cause of many local diseases in disorder of remote parts of the system, especially in the gastric organs, has led to the greatest improvement in the treatment of this, as well as in many other analogous affections. This indication is well followed by Mr. Curtis: in all cases of deafness, excepting those of an organic nature expressly local, his attention is directed to the state of the constitution; and many cases of what are vaguely termed *nervous* deafness, of several years' standing, have been perfectly relieved, by a course of calomel, purgatives, &c. assisted by appropriate local applications.

At the same time that we mention, that the author has taken advantage of the observations of those who have preceded him in this branch of practice, we must remark, that he has applied the principles above inculcated in an active and judicious manner; and the results of his experience appear to have been particularly favourable.

Histories of several cases of the different species of disease of the ear are adduced; but they are too concise, and are of no more value to the medical practitioner than general observations. In order that cases of disease may convey useful particular indications, they must be related in a more accurate and comprehensive manner.

